# Newcastle disease virus-based H5 influenza vaccine protects chickens from lethal challenge with a highly pathogenic H5N2 avian influenza virus

**DOI:** 10.1038/s41541-017-0034-4

**Published:** 2017-12-04

**Authors:** Jingjiao Ma, Jinhwa Lee, Haixia Liu, Ignacio Mena, A. Sally Davis, Sun Young Sunwoo, Yuekun Lang, Michael Duff, Igor Morozov, Yuhao Li, Jianmei Yang, Adolfo García-Sastre, Juergen A. Richt, Wenjun Ma

**Affiliations:** 1 Department of Diagnostic Medicine/Pathobiology, College of Veterinary Medicine, Kansas State University, Manhattan, KS USA; 2 Department of Microbiology, Icahn School of Medicine at Mount Sinai, New York, NY USA; 3 Global Health and Emerging Pathogens Institute, Icahn School of Medicine at Mount Sinai, New York, NY USA; 4 Innovation Team for Pathogen Ecology Research on Animal Influenza Virus, Department of Avian Infectious Disease, Shanghai Veterinary Research Institute, Chinese Academy of Agricultural Sciences, Shanghai, China; 5 Department of Medicine, Division of Infectious Diseases, Icahn School of Medicine at Mount Sinai, New York, NY USA; 6 Present Address: Shanghai Jiao Tong University, Shanghai, China

## Abstract

Since December 2014, Eurasian-origin, highly pathogenic avian influenza H5 viruses including H5N1, H5N2, and H5N8 subtypes (called H5N*x* viruses), which belong to the H5 clade 2.3.4.4, have been detected in U.S. wild birds. Subsequently, highly pathogenic H5N2 and H5N8 viruses have caused outbreaks in U.S. domestic poultry. Vaccination is one of the most effective ways to control influenza outbreaks and protect animal and public health. Newcastle disease virus (NDV)-based influenza vaccines have been demonstrated to be efficacious and safe in poultry. Herein, we developed an NDV-based H5 vaccine (NDV-H5) that expresses a codon-optimized ectodomain of the hemagglutinin from the A/chicken/Iowa/04-20/2015 (H5N2) virus and evaluated its efficacy in chickens. Results showed that both live and inactivated NDV-H5 vaccines induced hemagglutinin inhibition antibody titers against the H5N2 virus in immunized chickens after prime and booster, and both NDV-H5 vaccines completely protected chickens from lethal challenge with the highly pathogenic H5N2 A/turkey/Minnesota/9845-4/2015 virus. No clinical signs and only minimal virus shedding was observed in both vaccinated groups. In contrast, all mock-vaccinated, H5N2-infected chickens shed virus and died within 5 days post challenge. Furthermore, one dose of the live NDV-H5 vaccine also provided protection of 90% chickens immunized by coarse spraying; after exposure to H5N2 challenge, sera from vaccinated surviving chickens neutralized both highly pathogenic H5N1 and H5N8 viruses. Taken together, our results suggest that the NDV-based H5 vaccine is able to protect chickens against intercontinental highly pathogenic H5N*x* viruses and can be used by mass application to protect the poultry industry.

## Animal health: Protecting chickens from avian influenza

Vaccines based on Newcastle disease virus have proved efficacious in protecting chickens from H5 avian influenza strains. Avian influenza causes significant losses to the agriculture industry, indicating the importance of research into their control. A research collaboration of US and Chinese scientists, led by Kansas State University’s Wenjun Ma, have now produced vaccines based on Newcastle disease virus (NDV) — a platform easily modified to express immunity-stimulating proteins from other pathogens. Chickens vaccinated with the group’s vaccines survived a lethal dose of avian flu strain H5N2 and generated neutralizing antibodies cross-protective against other avian flu subtypes. The vaccine was easily applied by spraying the animals. The authors suggest that their data, in addition to previous studies, indicate that NDV could also be a useful vaccine platform for mammals such as humans.

## Introduction

Highly pathogenic avian influenza (HPAI) H5N8 viruses have spread globally to many countries; firstly, they were reported in Korea, subsequently in 2014 in China, Japan, Germany, United Kingdom, and Italy.^1,2^ A reassortant HPAI H5N2 virus that contains 5 gene segments (PB2, PA, HA, M, and NS) related to Eurasian HPAI H5N8 was found in Canada in November 2014.^3^ The US Department of Agriculture (USDA) confirmed HPAI H5N8 and H5N2 virus infections in wild birds in the state of Washington in December, 2014.^4^ The H5N8, and reassortant H5N2, and H5N1 viruses (altogether called H5N*x* viruses), in which the H5 gene belongs to clade 2.3.4.4, have been subsequently detected in 21 U.S. States, where 15 States reported outbreaks in domestic poultry and 6 States only in wild birds.^5^ The control of the 2015 HPAI outbreaks in the U.S. required euthanasia of approximately 49 million domestic birds (42 million chickens, 7 million turkeys) according to USDA reports.^6^ Both H5N2 and H5N1 viruses found in the U.S. are reassortant viruses, the latter one has only been found in the wild birds. The H5N2 virus contains PB2, PA, HA, M, and NS genes from the Eurasian HPAI H5N8 virus and the PB1, NP, and NA genes from North American low pathogenic avian influenza viruses;^7^ while the H5N1 virus contains PB2, HA, NP, and M genes from the Eurasian H5N8 virus and the PB1, PA, NA, and NS genes from North American low pathogenic avian influenza viruses.^8^ The HA genes of both HPAI H5N1 and H5N2 viruses are genetically related to the H5N8 virus earlier isolated in South Korea. The homology of H5 genes among HPAI H5N1, H5N2, and H5N8 viruses are more than 99% at the nucleotide level.^7^ Intercontinental H5N*x* viruses can infect wild birds without clinical signs but cause severe disease and death in domestic birds. The introduction of the HPAI H5N*x* viruses into U.S. domestic poultry in 2015 has resulted in significant economic losses for the poultry industry (estimated to be $4 billion in the U.S.) and international trade issues.

Although the culling or stamp out of infected poultry is an effective way to prevent the spread of HPAI viruses in isolated outbreaks, these efforts are compromised by violating poultry movement control and surveillance around outbreaks. In addition, there is a limited knowledge and capacity for safe and humane culling of millions of poultry and disposal. As the incidence of outbreaks within a country increases, animal health authorities can rapidly become overwhelmed through lack of resources and personnel. Therefore, a combination of culling and vaccination might be a better approach. Vaccination remains one of the most efficient methods to combat influenza infections. Since influenza viruses evolve rapidly over time, the closer the vaccine virus matches the circulating virus, the more protective the vaccine is.^9^ However, no commercial vaccines were a good match to the highly pathogenic H5N*x* viruses that caused the 2014-1015 outbreaks in the USA.

Newcastle disease caused by Newcastle disease virus (NDV) is an important disease of poultry for which live attenuated NDV vaccines are in wide use. Furthermore, NDV can be modified using reverse genetics and used as a vaccine platform to express one or more foreign antigens in order to develop bivalent vaccines against both NDV and other pathogens including influenza.^10,11^ NDV-vectored vaccines have DIVA (differentiating infected from vaccinated animals) capability for influenza and other pathogens because they express only one major antigen from target pathogen. Moreover, the attenuated NDV vaccine can be applied to large number of poultry through drinking water or aerosolization. For example, two NDV vaccines (Merck B1VAC and CLONEVAC-30) have been approved to be used by these application routes. NDV has been used as a vaccine vector for several pathogens infecting animals and humans;^12–15^ it has also been developed for cancer therapy.^16^ NDV LaSota strain is an avirulent virus and has been widely used as a vaccine in chickens.^17^ NDV-vectored influenza vaccines based on the LaSota strain have been demonstrated to be safe and efficacious against HPAI or LPAI virus challenge.^9,11,18–22^ One advantage of these recombinant vaccines is that they can be produced within four to six weeks, which is critical since influenza vaccine should be based on the circulating virus strain in the field.

In this study, a LaSota NDV-vectored H5 vaccine (NDV-H5) was developed based on the H5 gene of the HPAI A/chicken/Iowa/04-20/2015 (H5N2) virus. Subsequently, the efficacy of both live and inactivated NDV-H5 vaccines were evaluated in chickens by using the intramuscular immunization and challenging them with an HPAI A/turkey/Minnesota/9845-4/2015 (H5N2) virus. In addition, the efficacy of the live NDV-H5 vaccine was evaluated in chickens by using coarse spraying immunization and challenging them with the same HPAI H5N2 virus, and sera from vaccinated surviving birds were tested for neutralizing both HPAI H5N1 A/American green-winged teal/ Washington /195750/2014 and H5N8 A/gyrfalcon/ Washington /41088/2014 viruses.

## Results

### Generation and characterization of the NDV-H5 vaccine candidate

**Fig. 1:**
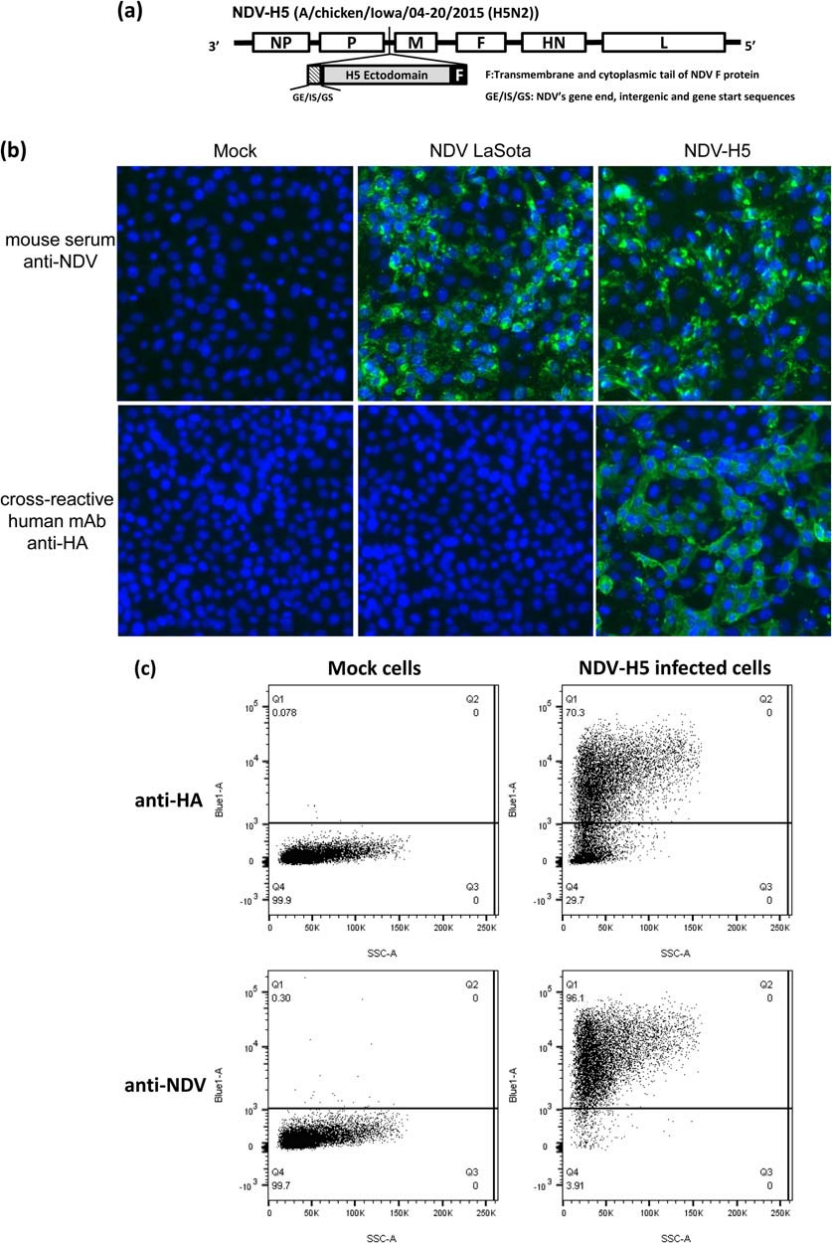
Schematic design of recombinant NDV-H5 constructs and characterization of recombinant NDV-H5 virus in infected Vero cells. **a** The chicken codon-optimized ectodomain of hemagglutinin gene from the A/chicken/Iowa/04-20/2015 (H5N2) was fused with the transmembrane and cytoplasmic tail of fusion (F) protein of NDV-LaSota strain, and cloned into the P and M junction of the NDV antigenomic cDNA. The H5 ectodomain was placed under the control of a set of NDV gene start (GS) and gene end (GE) transcription signals directing its expression as a separate mRNA. **b**, **c** H5 protein was expressed and detected in NDV-H5 virus infected Vero cells using an HA human-monoclonal antibody. NDV proteins were detected in Vero cells infected with the NDV-H5 or NDV LaSota virus using mouse polyclonal NDV serum by immunofluorescence and flow cytometry assays

The codon-optimized ectodomain of an H5 HA based on the H5N2 A/chicken/Iowa/04-20/2015 virus was cloned into the NDV vector and confirmed by sequencing. The full-length cDNA clone carrying the complete antisense genome of the NDV LaSota vaccine strain with the H5 HA ectodomain part fused with the transmembrane and cytoplasmic tail of the NDV F protein (Fig. 1a) were co-transfected with the supporting NDV plasmids (NP, P, and L) into A549 cells. The recombinant NDV-H5 virus was rescued and amplified in SPF chicken eggs as described previously.^23^ Our former study demonstrated that this chimeric strategy to generate recombinant NDV H5 and H7 viruses, i.e., influenza virus HA ectodomain fused to the NDV F protein transmembrane and cytoplasmic regions, results in efficient incorporation of the recombinant HA into the viral particle.^18^

To determine the HA expression by the recombinant NDV-H5 virus, we conducted immunofluorescence and flow cytometry assays using specific antibodies targeting the influenza HA and NDV antigens. Results revealed that specific green fluorescence was detected in Vero cells infected with the NDV-H5 virus using an anti-HA monoclonal antibody, but not in those infected with the wild type NDV LaSota virus (Fig. 1b); however, NDV antigens could be detected in Vero cells infected with either the NDV-H5 or the control NDV LaSota virus (Fig. 1b). Expression of HA by the recombinant NDV-H5 virus was further confirmed by flow cytometry assay (Fig. 1c). All results indicated that the recombinant NDV-H5 virus successfully expressed the H5 HA protein and could be used as a vaccine candidate for further experiments.

### Both live and inactivated NDV-H5 are immunogenic in chickens

**Fig. 2:**
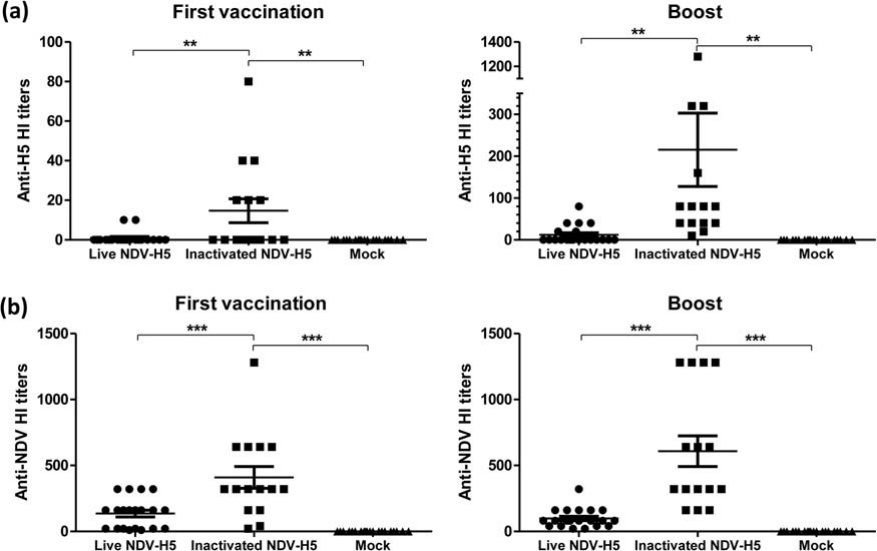
Serum HI titers against H5 and NDV in mock-vaccinated and vaccinated chickens after two weeks of prime and booster. **a** Anti-H5 HI titer in each individual bird in each group of immunized chickens with indicated vaccine at 2 weeks post first vaccination and 2 week post boost. **b** Anti-NDV HI titer in each individual bird in each group of immunized chickens with indicated vaccine at 2 weeks post first vaccination and 2 week post boost. (Round dot: live NDV-H5; square: inactivated NDV-H5; triangle: mock-vaccinated)

To determine immunogenicity of live and inactivated NDV-H5 vaccines in chickens, both anti-H5 and anti-NDV antibody levels were tested by HI assay prior to vaccination, at booster vaccination (2 weeks post first vaccination) and before challenge. No antibodies against H5 and NDV were detected in all experimental chickens prior to vaccination. Detectable HI titer (1:10) against the H5 virus was detected only in 2 out of 20 chickens immunized with the live NDV-H5 virus at 2 weeks post the first vaccination, while 15 out of 20 birds in this group showed seroconversion (HI titer ranges from 10 to 80) after 2 weeks post booster (Fig. 2a). In contrast, 6 out of 15 chickens immunized with the inactivated NDV-H5 vaccine seroconverted at 2 weeks post the first vaccination with an HI titer ranging from 20 to 80 against the H5 virus; a significantly higher HI titer against the H5 virus was found in all birds (HI titer ranges from 10 to 1280) after 2 weeks post booster (Fig. 2a). Regarding the HI titers against NDV LaSota strain virus, all vaccinated birds with either live or inactivated NDV-H5 vaccine seroconverted at 2 weeks post the first vaccination with titers ranging from 10 to 1280. However, the inactivated NDV-H5 induced a significantly higher titer in immunized chickens than the live NDV-H5 vaccine (Fig. 2b). Two weeks post booster, the inactivated NDV-H5 vaccinated chickens showed an increased antibody titer against NDV LaSota ranging from 160 to 1280. In contrast, the chickens vaccinated with the live NDV-H5 virus displayed a similar or even decreased HI titer in most of the birds after booster vaccination; only on 6 birds which had a low titer (10–20) after the first vaccination, an increased HI titer was detected (Fig. 2b). These results indicated that live and inactivated NDV-H5 vaccines were immunogenic in chickens, whereas the inactivated NDV-H5 vaccine was more immunogenic in chickens than the live NDV-H5 vaccine.

### Both live and inactivated NDV-H5 vaccines are able to protect chickens from lethal challenge

**Fig. 3:**
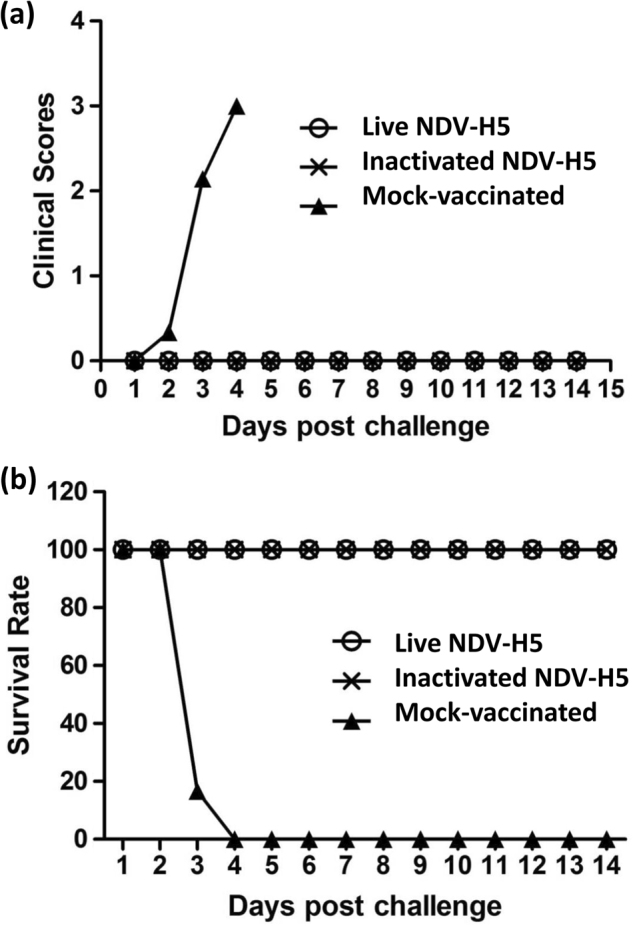
Clinical scores and survival rate of vaccinated and mock-vaccinated chickens after challenge. **a** Clinical scores were given in each group of chickens based on the clinical symptoms. The scores range from 0 to 3. (0: no clinical sign; 1: mild clinical signs; 2: severe clinical signs; 3: dead). Only the mock-vaccinated chickens showed clinical symptoms. **b** Survival rates of mock-vaccinated and vaccinated chickens were calculated daily. Both live and inactivated NDV-H5 vaccinated chickens survived and none of mock-vaccinated chickens survived from the challenge

Chickens immunized with either the live or inactivated NDV-H5 vaccine, similar as the mock-vaccinated chickens didn’t show any clinical symptoms during the vaccination period. At 2 or 3 days post challenge (dpc) with the HPAI H5N2 virus, all mock-vaccinated chickens started to show clinical signs, such as ruffled feathers, facial edema, depression, swollen and cyanotic wattles and combs, petechial hemorrhages on unfeathered skin and larger subcutaneous shank hemorrhages, and central nervous system (CNS) symptoms, and sudden death. Four birds (pre-assigned) were scheduled to be necropsied on 3 dpc in order to investigate virus replication; one of them was found death on 2 dpc, and the other 3 displayed severe symptoms when necropsied on 3 dpc. The remaining 11 birds in the mock-vaccinated group were either found dead or were humanely euthanized due to severe disease on 3 or 4 dpc. The severity of disease was scored according to clinical symptoms as shown in Fig. 3a. In contrast to mock-vaccinated birds, no immunized animals with either the live or inactivated NDV-H5 vaccine showed any clinical symptoms throughout the observation period of 14 days; all survived the lethal H5N2 challenge. These results indicated that both live and inactivated NDV-H5 vaccines were able to provide 100% protection in chickens against lethal H5N2 virus challenge (Fig. 3b).

### Both live and inactivated NDV-H5 vaccines reduce viral shedding and systemic replication

To investigate whether vaccination can block virus shedding and virus replication in organs, virus titer was determined in oropharyngeal and cloacal swabs and various organs. Nine out of 15 mock-vaccinated chickens shed virus through the oropharyngeal cavity at 1 dpc, and 15 out of 15 birds at 3 dpc. No virus was detected in cloacal samples collected from mock-vaccinated chickens at 1 dpc, but virus shedding was detected in 2 out of 8 birds in cloacal swabs at 3 dpc. In contrast, virus was only found in oropharyngeal swabs collected from 1 out of 20 chickens immunized with the live NDV-H5 and 2 out of 15 birds immunized with the inactivated NDV-H5 vaccine at 1 dpc. No virus was detected in cloacal swabs and in oropharyngeal swabs collected at any other days in both live and inactivated NDV-H5 vaccinated chickens (Table 1A).

**Table 1:** Viral titers in collected swab and tissue samples from experimental chickens. (A) Viral titers in oropharyngeal and cloacal swabs collected from vaccinated and mock-vaccinated chickens at 1, 3, 5, 7, and 9 days post challenge; (B) Viral titers in different tissues collected from vaccinated or mock-vaccinated chickens at different days post challenge

	1 dpc		3 dpc		5 dpc		7 dpc		9 dpc	
	Oropharyngeal	Cloacal	Oropharyngeal	Cloacal	Oropharyngeal	Cloacal	Oropharyngeal	Cloacal	Oropharyngeal	Cloacal
(A)										
Live NDV-H5	1.7^a^ (1/20)^b^	<1.0	<1.0	<1.0	<1.0	<1.0	<1.0	<1.0	<1.0	<1.0
Inactivated NDV-H5	2.5 ± 0^a^ (2/15)^b^	<1.0	<1.0	<1.0	<1.0	<1.0	<1.0	<1.0	<1.0	<1.0
Mock-vaccinated	2.43 ± 0.18^a^ (9/15)^b^	<1.0	3.18 ± 0.31^a^ (8/8)^b^	1.7 ± 0^a^ (2/8)^b^	N/A	N/A	N/A	N/A	N/A	N/A

	Lung		Spleen		Bursa	
	3 dpc	5 dpc	3 dpc	5 dpc	3 dpc	5 dpc
(B)						
Live NDV-H5	<1.0	<1.0	<1.0	<1.0	<1.0	<1.0
Inactivated NDV-H5	<1.0	<1.0	<1.0	<1.0	<1.0	<1.0
Mock-vaccinated	3.7 ± 0.35^a^ (7/8)^b^	N/A	2.86 ± 0.46^a^ (5/8)^b^	N/A	3.67 ± 0.36^a^ (7/8)^b^	N/A

*N/A* none applicable. Chickens were dead before the samples collection days

^a^ Log_10_ TCID_50_/mL

^b^ Positive bird numbers out of total numbers

Virus replication was determined in lung, spleen and bursa fabricii collected at 3 and 5 dpc. Virus was detected in all three tissues (7/8 from lung, 5/8 from spleen, and 7/8 from bursa) collected from mock-vaccinated chickens at 3 dpc. In contrast, no virus was found in these three organs collected from chickens immunized with either the live or inactivated NDV-H5 vaccine at 3 and 5 dpc (Table 1B). These results indicate that both live and inactivated NDV-H5 vaccines are able to reduce virus shedding and systemic replication in chickens.

### Both live and inactivated NDV-H5 vaccines protect immunized chickens from pathological lesions after challenge

**Fig. 4:**
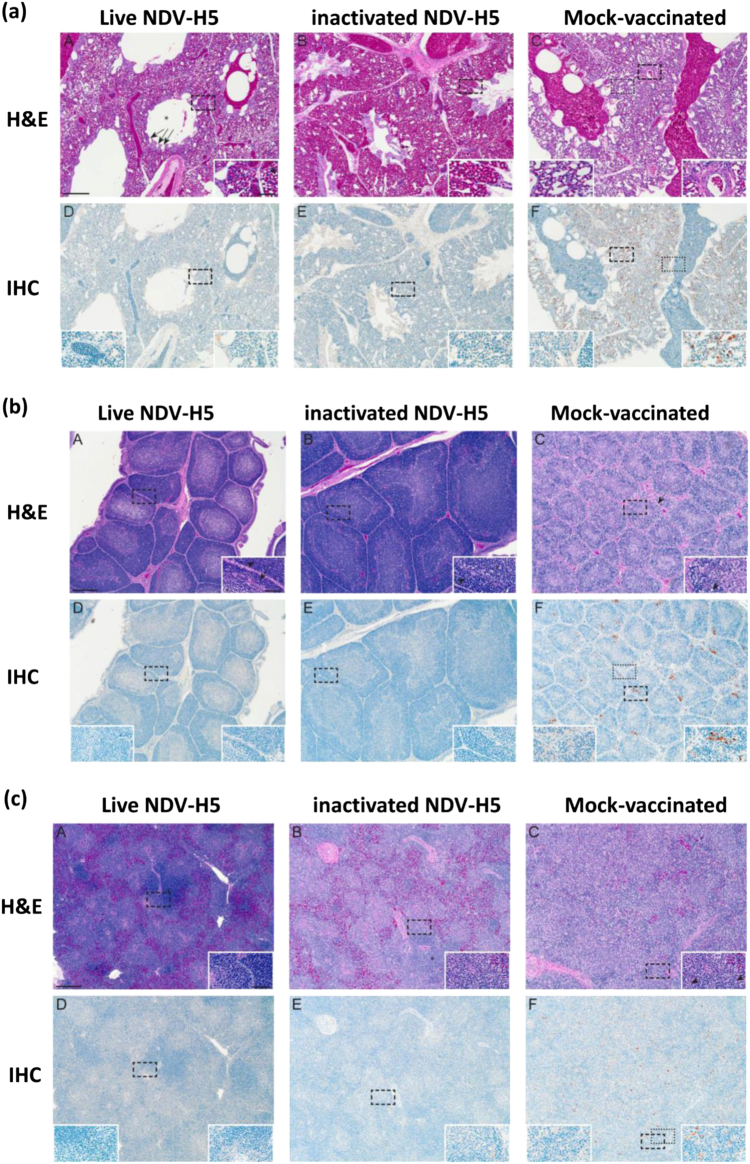
Histopathology and immunochemistry panel of lung, spleen, and bursa of vaccinated and mock-vaccinated chickens at 3 dpc. **a** H&E and IHC staining in the lungs of chickens that were vaccinated or mock-vaccinated as indicated. **b** H&E and IHC staining in the bursa of birds that were vaccinated or mock-vaccinated as indicated. **c** H&E and IHC staining in the spleens of birds that were vaccinated or mock-vaccinated as indicated

Histopathological (H&E) and immunohistological (IHC) analysis of lung, spleen and bursa fabricii from vaccinated and mock-vaccinated chickens challenged with the HPAI H5N2 virus revealed Influenza A virus-specific histopathology and positive IHC only in the mock-vaccinated chickens (Fig. 4). Mock-vaccinated birds had organ histopathological scores of 2 or 3, while the vaccinated birds with either live or inactivated NDV-H5 vaccine predominantly received scores of 0 (Table 2). In the lungs of mock-vaccinated birds, the parabronchial units were more affected than the other larger airways. Tracheal and bronchial cilia were still intact but their epithelium was multifocally hyperplastic and there was mild to moderate predominantly lymphohistiocytic subepithelial inflammation. These foci had scattered influenza positive cells. The main feature of the mock-vaccinated birds’ lungs was a nearly diffuse heterophilic and lymphohistiocytic interstitial pneumonia accompanied by multifocal necrosis of infundibular and atrial epithelial cells and perivascular lymphocyte accumulation (Fig. 4a). In mock-vaccinated chickens, there was diffuse moderate to severe lymphocytolysis within the Bursa of Fabricius follicles, both cortical and medullary, generally unaccompanied by inflammation (Fig. 4b) as well as nearly diffuse loss of splenic white pulp with marked infiltration of heterophils and pigmented macrophages (Fig. 4c). Both organs were positive for influenza NP antigen (Figs. 4b, c). In general, the chickens from both the live and inactivated vaccine groups had no histopathological lesions. Two birds from the live NDV-H5 and inactivated NDV-H5 vaccine groups, respectively, received lung scores of 1. The one from the live NDV-H5 group had mild, multifocal subepithelial inflammation in its bronchi and the bird from the inactivated NDV-H5 group had a mild infiltration of leukocytes, predominantly histiocytes, in its parabronchial septae. Additionally, occasional cytoplasm-only positive labeling for influenza NP antigen was observed in the tracheal and large airway epithelia in several vaccinated birds, mainly in scattered histiocytes. This is in contrast to the nuclear and cytoplasmic distribution of influenza antigen found in the mock-vaccinated birds. Vaccinated birds, regardless of vaccine type, exhibited a range of histological variance in the follicles of their bursa of Fabricius that was considered to be within normal range for 7-week-old chickens. Likewise, the prominence of splenic white pulp varied for the vaccinated birds unrelated to vaccine type.

**Table 2:** Histopathology scores of tissues collected from vaccinated and mock-vaccinated chickens at 3 and 4/5 dpc

	3 dpc			4/5^a^ dpc		
	Spleen	Bursa	Lung	Spleen	Bursa	Lung
Live NDV-H5	0 (0/5)	0 (0/5)	0.2 ± 0.22 (1/5)^b^	0 (0/5)	0 (0/5)	0 (0/5)
Inactivated NDV-H5	0 (0/4)	0 (0/4)	0.25 ± 0.25 (1/4)^b^	0 (0/4)	0 (0/4)	0 (0/4)
Mock-vaccinated^c^	2.8 ± 0.2 (5/5)^d^	2.6 ± 0.24 (5/5)^d^	2.8 ± 0.2 (5/5)^d^	3 (1/1)^c^	3 (1/1)^c^	2 (1/1)^c^

^a^ For the mock-vaccinated group, the tissues were collected from birds on 4 dpc; and for both vaccine groups, they were collected on 5 pdc

^b^ The sample was positive in pathology but negative in IHC

^c^ Three of the mock birds’ tissue sets could not be scored because interpretation of histopathology was hampered by autolysis. Sample set collected at 4 dpc

^d^ Positive bird numbers out of total numbers

### One dose of the live NDV-H5 vaccine through coarse spraying immunization protects 90% chickens from the HPAI H5N2 virus challenge

**Fig. 5:**
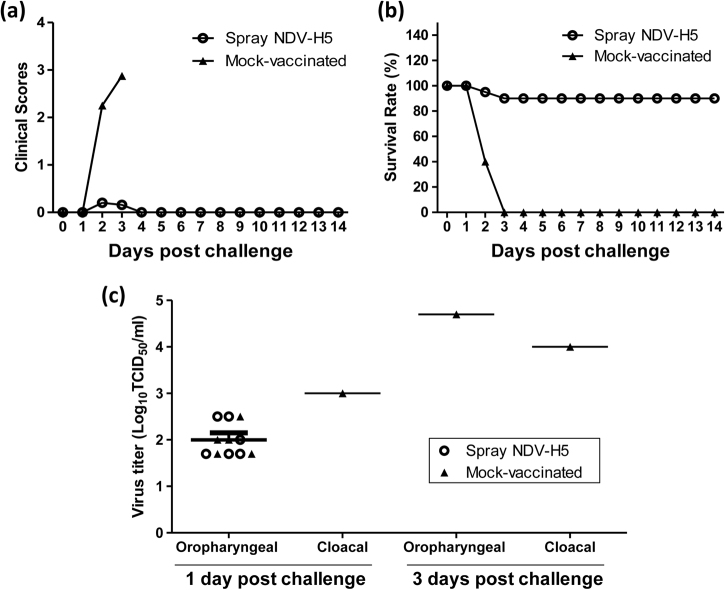
Clinical scores, survival rate and virus shedding of spray vaccinated and mock-vaccinated chickens after challenge. **a** Clinical scores were given in each group of chickens based on the clinical symptoms. The scores range from 0 to 3. (0: no clinical sign; 1: mild clinical signs; 2: severe clinical signs; 3: dead). All mock-vaccinated chickens and only 2 vaccinated birds showed clinical symptoms. **b** Survival rates of mock-vaccinated and vaccinated chickens were calculated daily. Eighteen NDV-H5 vaccinated chickens survived and none of mock-vaccinated chickens survived from the challenge. **c** Viral titers in oropharyngeal and cloacal swabs collected from vaccinated and mock-vaccinated chickens at 1 and 3 days post challenge

None of the birds used in this study had the detectable anti-H5 titers prior to vaccination. Only 1 out of 20 chickens immunized with a single dose of live NDV-H5 by coarse spraying had the detectable anti-H5 HI titer (1:20) at 3 weeks post vaccination. After challenge with the HPAI H5N2 virus, mock-vaccinated chickens showed severe clinical disease. Seventeen of 20 mock-vaccinated birds were found dead at 2 or 3 dpc, and the remaining 3 birds were humanely euthanized due to severe disease at 3 dpc (Fig. 5a, b). Only 1 out of 20 vaccinated chickens by coarse spraying was found dead at 2 dpc and another one was found dead at 3 dpc, while the remaining birds survived until the end of the experiment (14 dpc) without clinical symptoms (Fig. 5a, b). All surviving birds seroconverted with an HI titer ranging from 20 to 320 (geometric mean of HI titer is 71) against the H5N2 virus.

At 1 dpc, no virus was detected in cloacal swabs collected from vaccinated chickens, while 6 of 20 oropharyngeal swabs were positive by virus isolation. The virus titer ranged from 10^1.7^ to 10^2.5^ TCID_50_/mL. In contrast, virus was isolated from 5 oropharyngeal swabs (virus titers ranged from 10^1.7^ to 10^2.5^ TCID_50_/mL) and 1 cloacal swab (virus titer is 10^3.0^ TCID_50_/mL) collected from 20 mock-vaccinated chickens at the same date (Fig. 5c). At 3 dpc, virus was detected in oropharyngeal (virus titer is 10^4.7^ TCID_50_/mL) and cloacal swabs (virus titer is 10^4.0^ TCID_50_/mL) collected from one available mock-vaccinated birds (Fig. 5c); in contrast, no virus was detected in either oropharyngeal or cloacal swabs collected from the remaining 18 vaccinated birds at 3, 5, 7, and 9 dpc (Fig. 5c).

To investigate whether the anti-H5N2 sera from surviving birds are able to neutralize other HPAI H5 viruses, micro-neutralization (MN) assays were performed to neutralize both HPAI H5N1 A/American green-winged teal/Washington/195750/2014 and H5N8 A/gyrfalcon/ Washington /41088/2014 viruses. All 18 surviving vaccinated chickens had MN antibody titers against both HPAI H5N1 and H5N8 viruses ranging from 80 to 1280, with geometric mean of MN titers of 235 and 387 against the H5N1 and H5N8 viruses, respectively. Our results indicate that immunization of one dose of the live NDV-H5 vaccine by coarse spraying can provide 90% protection in vaccinated chickens, and significantly reduces virus shedding.

## Discussion

Eurasian-origin HPAI H5N8 virus has spread worldwide with migration of wild birds, posing a significant threat to the poultry industry. This H5N8 can readily reassort with locally endemic low pathogenic avian influenza viruses to generate novel viruses; the reassortant HPAI H5N1 and H5N2 viruses detected in the USA are good examples. The reassortant H5N2 and original Eurasian H5N8 viruses have caused outbreaks in U.S. domestic birds. In order to control outbreaks of HPAI worldwide, hundreds of millions of poultry have been slaughtered to prevent the spread of the virus. The development of safe and efficacious vaccines against HPAI viruses is urgently needed to protect the poultry industry, export markets and our food supply.

In the present study, we have developed an NDV-H5 vaccine based on the NDV LaSota vaccine strain, and demonstrated that both inactivated and live NDV-H5 vaccines are able to protect chickens against a lethal HPAI H5N2 virus challenge. This was shown by protection of vaccinated chickens against clinical symptoms and mortality, by blocking virus replication in various organs and by significantly reducing viral shedding. Although both vaccines were given through the same IM route, the antibody levels of anti-HA and anti-NDV antibodies in chickens prime immunized with the inactivated NDV-H5 vaccine were much higher than in those immunized with the live NDV-H5 vaccine. This could be due to the fact that (i) the inactivated NDV-H5 vaccine contained more virus antigen per dose and an adjuvant which increases the immunogenicity of vaccines^24^ in contrast to the live vaccine formulation, and (ii) the live NDV-H5 most likely does not replicate well in chickens through the IM immunization route as replication of the NDV LaSota strain is mainly in the mucosa. The anti-HA specific antibody levels in birds increased in both live and inactivated NDV-H5 vaccines after booster; however, the anti-NDV antibody levels decreased in the live NDV-H5 vaccine group after booster, whereas the titer in inactivated NDV-H5 vaccine group increased when compared to the titer after the first vaccination. This may indicate that pre-existing NDV antibodies, such as maternal antibodies, could affect the immune responses after live NDV vaccination,^25,26^ whereas pre-existing NDV antibodies had almost no effect on the booster vaccination using the inactivated vaccine. Similar results have been presented in previous studies where application of a live NDV vaccine could not induce the same high levels of NDV-specific antibody levels as the inactivated vaccine when maternal antibodies were present in the birds.^25,26^ However, the live NDV-H5 vaccine application was still able to provide 100% protection of chickens from the lethal H5N2 challenge. A previous study showed that a live recombinant LaSota NDV-H5 vaccine (rNDV-LS/AI-H5) is efficacious in protecting chickens with high NDV-specific maternal antibodies against challenge with a virulent HPAI H5 virus and a velogenic NDV strain.^27^ These results suggest that the live NDV-H5 vaccine could also overcome the interference of existing NDV-specific maternal antibodies. This question needs to be investigated in future studies.

Furthermore, we have performed a study using a Pump-up Sprayer to massively apply the live NDV-H5 vaccine via coarse spraying in order to test the protective efficacy using a labor cost-effective immunization strategy. Results showed that the engineered live NDV-H5 vaccine is able to provide 90% protection to chickens through one single dose of spray vaccination. It should be noted that failure to provide complete protection in immunized birds is probably due to following two reasons: (1) immunization is taken up unevenly as two chickens displayed severe symptoms at 2-3 dpc, indicating that these birds probably were not exposed to the vaccine; (2) a Pump-up Sprayer used for vaccination produced droplets of size approximately 300 μ, which will affect vaccine inhalation through nasal cavity and mucous absorption through the eyes. We suggest that protection efficacy of the NDV-H5 vaccine could be improved if an ideal instrument for coarse spray immunization are designed to produce correct size of droplets. Future research avenues will investigate if this is the case. In any case, our studies here indicate that the newly developed NDV-H5 vaccine could be used to combat outbreaks caused by the intercontinental H5N*x* viruses.

It should be noted that the vaccination regimen used, i.e., timing between vaccinations (2 weeks between prime and boost for the intramuscular immunizations) and challenge is relatively short (2–3 weeks) in both our studies. This indicates that the vaccine is effective even after a few weeks post-vaccination. However, this timing does not allow the evaluation of long term protection. In order to evaluate how long the immune responses induced by the developed NDV-H5 vaccine will last, challenges should be conducted later such as 8 or more weeks post vaccination. Therefore, additional studies are needed to determine long-term protection of the developed NDV-H5 vaccine. HPAI H5 viruses cause severe impacts not only on chickens, but also on turkeys and domesticated waterfowls. We have demonstrated that the developed NDV-H5 vaccine is able to provide efficient protection in immunized chickens. The efficacy of the NDV-H5 vaccine in other species, such as turkeys, ducks and geese, will be evaluated in our future studies.

It is important to note that we are able to produce the recombinant NDV-vectored influenza vaccine with DIVA capability within 4–6 weeks after we receive the HA sequence of a particular influenza strain. This is important since influenza vaccines have to be updated regularly based on the circulating or newly emerging virus strain. Therefore, an NDV vector-based influenza vaccine could be produced as fast as a conventional inactivated influenza virus vaccine, with the advantage that the NDV-based vaccine can be applied by coarse spraying. A previous study has shown that an NDV-vectored influenza H5 vaccine is able to induce high titer of serum neutralizing antibodies against HPAI H5 virus in a nonhuman primate model, the African green monkey.^28^ This indicates that NDV-vectored influenza vaccines most likely could also be used to vaccinate mammalian species against influenza viruses. In this aspect, it should be noted that a NDV-vectored rabies vaccine has been shown to be safe, highly immunogenic, to provide long-lasting protection in dogs and cats.^29^ All these data indicate that the NDV-vectored influenza vaccines might be efficacious in various mammalian hosts including humans against influenza infection. Since NDV is not a mammalian pathogen, this approach should be safe in mammals.

## Methods

### Ethics statements

All animal studies were approved and carried out in strict accordance to the recommendations in the guidelines of the Institutional Animal Care and Use Committee at Kansas State University, an AAALAC institution. All researches related to highly pathogenic H5 viruses were performed in biosafety level 3 laboratory and facilities (BSL3) in the Biosecurity Research Institute at Kansas State University.

### Viruses, cell culture, and eggs

HPAI H5 viruses including A/chicken/Iowa/04-20/2015 (H5N2), A/turkey/Minnesota/9845-4/2015 (H5N2), A/American green-winged teal/Washington/195750/2014 (H5N1) and A/gyrfalcon/Washington/41088/2014 (H5N8) were kindly provided by the National Veterinary Services Laboratories (NVSL) at Ames, IA and propagated in ten-day-old specific-pathogen-free (SPF) embryonated chicken eggs. Mardin-Darby Canine Kidney Cells (MDCK) were maintained in Minimum Essential Medium (MEM) supplemented with 5% fetal bovine serum (FBS) (HyClone; Logan UT), 1x L-glutamine (Invitrogen, Carlsbad, CA), 1x MEM vitamins (Invitrogen; Carlsbad, CA), and 1x antibiotics (Invitrogen, Carlsbad, CA) under 37 °C with 5% CO_2_. Adenocarcinomic human alveolar basal epithelial cells (A549) were used for transfection of NDV anti-genome cDNA with NDV supporting plasmids expressing NDV N, P, and L to generate recombinant NDV viruses. African green monkey kidney (Vero) and A549 cells were cultured in Dulbecco’s Modified Eagle Medium (DMEM) maintained and supplemented with 10% FBS and other reagents as the MDCK cells described above. Virus titers were determined on 96-well MDCK cells and TCID_50_ per milliliter was calculated by the Reed and Muench method.^30,31^

### Vaccine construction and preparation

Newcastle disease virus-vectored (NDV-vectored) vaccine was constructed by expressing the codon optimized ectodomain of the H5 hemagglutinin (HA) gene from the A/chicken/Iowa/04-20/2015 (H5N2) virus (Genbank accession number KR492974.3). The multiple basic cleavage site (RERRRKR/GLF) of H5 was replaced with a monobasic cleavage site (ESR/GLF). The optimized sequence coding the ectodomain of the HA was fused in frame to the sequence coding the trans-membrane and cytoplasmic tail of the NDV fusion protein and subsequently cloned between the P and M genes of a full length cDNA of the NDV vaccine strain LaSota as described previously.^18^ Flanking NDV’s gene end (GE), intergenic (IS), and gene start (GS) regulatory sequences were inserted to ensure recognition as an additional viral gene. A Kozak sequence was inserted upstream of the start codon for optimal initiation of translation (Fig. 1a). The recombinant NDV virus expressing the H5 HA (NDV-H5) was rescued by reverse genetics, then amplified in SPF embryonated chicken eggs for further experiments.

To verify if the H5 HA protein was expressed by the recombinant NDV-H5 virus, Vero cells were infected with the NDV-H5 virus and fixed at 24 hpi, and immunofluorescence assay was performed to detect HA protein using a human monoclonal antibody CR9114^32^ that recognizes a conserved epitope in the stalk domain of the HA from influenza A and B viruses. To detect NDV antigens, we used a mouse-origin anti-NDV polyclonal serum prepared by pooling the serum of 5 mice infected with the wild type NDV, LaSota strain. Fluorescently labeled anti-human IgG and anti-mouse IgG (Invitrogen) were used as secondary antibodies. Flow cytometry was used to confirm HA protein expression by NDV-H5 virus infected Vero cells using the same anti-HA monoclonal antibody. The wild-type NDV LaSota strain was used as a control for both assays.

The amplified recombinant NDV-H5 virus was concentrated by ultracentrifugation using a 30% sucrose cushion. The concentrated virus was titrated and inactivated by the UV for 1 h. The inactivated virus was mixed with 15% water-in-oil adjuvant (ISA 70, Seppic) to make inactivated NDV-H5 vaccine. Inactivated NDV-H5 vaccine contains the equivalent to 10^7^ TCID_50_ of NDV-H5 virus per dose prior to inactivation.

### Chicken experiments

To evaluate the efficacy of the developed NDV-H5 vaccine, two experiments using two-week-old SPF White Leghorn chickens (purchased from Charles River Laboratories) were performed. In the first experiment, the NDV-H5 vaccine was administered through the intramuscular (i.m.) route, not through the oculonasal route, because our pervious study showed that the antibody levels of chickens vaccinated intramuscularly were higher than those of chickens vaccinated oculonasally.^18^ Fifty chickens were randomly separated to three groups. Twenty chickens in the group 1 were intramuscularly immunized with 5 × 10^6^ TCID_50_ of live NDV-H5 vaccine per bird in 200 μl. Fifteen chickens in the group 2 were immunized with the inactivated NDV-H5 vaccine (10^7^ TCID_50_ in 200 μl per chicken) through the i.m. route. The remaining 15 chickens were intramuscularly inoculated with 200 μl of phosphate-buffered saline (PBS) as mock-vaccinated controls. Two weeks after the first vaccination, chickens were boosted with the same vaccine using the same procedure as that of the first vaccination. Two weeks after the booster, all chickens were oculonasally challenged with 10^6^ TCID_50_ of an HPAI A/turkey/Minnesota/9845-4/2015 (H5N2) virus in 200 μl. Chickens were bled to collect serum samples prior to each vaccination and challenge. Oropharyngeal and cloacal swabs were collected on 0, 1, 3, 5, 7, 9, 11, and 13 days post challenge (dpc). Clinical signs were monitored daily. Five chickens in the live NDV-H5 immunization group, 4 chickens in the NDV-H5 inactivated vaccine immunization group, and 4 chickens in mock-vaccinated control group were predesignated to be euthanized on 3 and 5 dpc. When a necropsy designated bird was found dead prematurely or if a bird met study end point criteria for euthanasia, they were necropsied at that time point instead. At necropsy the lungs, spleen, and the bursa of Fabricius were collected from each bird. A piece from each organ was frozen for virological analysis and the remainder was placed in 10% neutral buffered formalin for histopathological analysis. The remaining birds in each group were monitored for 14 days in order to determine vaccine efficacy. At 14 dpc, all surviving birds were euthanized. Sera were collected from all necropsied chickens, and hemagglutination inhibition (HI) assay was performed to determine seroconversion.

In the second experiment, 40 two-week-old chickens were divided into two groups. Twenty chickens were immunized with the live NDV-H5 vaccine by using a coarse sprayer (Pump-up Sprayer #28584, Valley Vet Supply, Marysville, Kansas) to spray chicken feathers and bodies when they grouped together in a plastic box. Each bird was predicted to get 10^6^ TCID_50_ of the live NDV-H5 virus through contacting the aerosol produced by the coarse spraying. The remaining 20 chickens were sprayed with PBS and used as mock-vaccinated controls. Each chicken was challenged with 10^6^ TCID_50_ of the HPAI A/turkey/Minnesota/9845-4/2015 (H5N2) as described above at 21 days post vaccination. Chickens were bled to collect serum samples before vaccination and challenge. In addition, the challenged chickens were observed daily to monitor clinical signs and mortality. Oropharyngeal and cloacal swabs were collected at 0, 1, 3, 5, 7, and 9 dpc to determine viral shedding. Serum samples were collected from all surviving chickens prior to euthanasia at 14 dpc to perform the micro-neutralization assay to determine cross-reactions with both HPAI H5N1 A/American green-winged teal/WA/195750/2014 and H5N8 A/gyrfalcon/WA/41088/2014 viruses based on the standard protocol.

### Histopathology and immunohistochemistry

Standard hematoxylin and eosin (H&E) slide staining for the collected tissues was performed and influenza nucleoprotein (NP) was detected in the tissues by immunohistochemistry (IHC) assay using a Rabbit Anti-H1N1 NP polyclonal antibody (GenScript, NJ) as described in our prior study.^18^ H&E stained tissues and immunohistochemistry were reviewed by a veterinary pathologist in a blinded fashion. Histopathology for all necropsied birds was semi-quantitatively scored for each organ on a scale of 0–3 unless prevented by autolysis. For all tissues a score of 0 was within normal limits. For the lungs: score 1 was increased interstitial lymphocytes and histiocytes, score 2 was mild to moderate multifocal heterophilic and lymphistiocytic interstitial pneumonia; multifocal, mild to moderate lymphohistiocytic bronchitis and tracheitis; multifocally, low numbers of perivascular lymphocytes and score 3 was moderate to severe, diffuse heterophilic and lymphohistiocytic interstitial pneumonia; multifocal to nearly diffuse moderate tracheitis and bronchitis. For the spleen: score 1 was occasional foci of splenic necrosis in the white pulp, score 2 was multifocal lymphoid depletion, splenic necrosis, and mild histiocytic and heterophilic splenitis, and score 3 was nearly diffuse lymphoid depletion, splenic necrosis, and moderate to severe histiocytic and heterophilic splenitis with marked hyperplasia of phagocytic macrophage cells. For the bursa of Fabricius: score 1 was cortical or medullary lymphoid depletion and/or mild interstitial or epithelial inflammation, score 2 was moderate follicular lymphocytolysis, cortical and medullary optionally accompanied by inflammation, multifocal loss of distinction between medulla and cortex, and score 3 was severe follicular lymphocytolysis, cortical and medullary, loss of cortical medullary distinction, pooling of proteinaceous material to formation of cysts in the medulla, optionally accompanied by inflammation. IHC results were recorded as presence or absence of influenza antigen in each organ in predesignated 3 dpc birds only. All microscopic images were captured with a BX46 light microscope equipped with a DP25 camera (Olympus; Tokyo, Japan) using CellSens Standard version 1.12 (Olympus) then further color calibrated using ChromaCal software version 2.5 (Datacolor Inc., Lawrenceville, NJ) as per manufacturer’s instructions.

### Statistical analysis

Virus titers and antibody titers among groups were analyzed using One-way ANOVA in GraphPad Prism version 6.0 (Graph-Pad Software Inc., CA). Those response variables were subjected to comparisons for all pairs by using the Tukey–Kramer test. Pairwise mean comparisons between vaccinated and mock groups were made using the Student *t*-test. A *p* value of ≤0.05 was considered as a significant difference.

### Data availability statements

The datasets generated during and/or analyzed during the current study are available from the corresponding authors on reasonable request.

## References

[1] Lee YJ (2014). Novel reassortant influenza A(H5N8) viruses, South Korea, 2014. Emerg. Infect. Dis..

[2] Smith GJ, Donis RO (2015). Nomenclature updates resulting from the evolution of avian influenza A(H5) virus clades 2.1.3.2a, 2.2.1, and 2.3.4 during 2013–2014. Influenza Other Respir. Viruses.

[3] Pasick J (2015). Reassortant highly pathogenic influenza A H5N2 virus containing gene segments related to Eurasian H5N8 in British Columbia, Canada, 2014. Sci. Rep..

[4] Jhung MA, Nelson DI (2015). Outbreaks of avian influenza A (H5N2), (H5N8), and (H5N1) among birds—United States, December 2014–January 2015. MMWR.

[5] Highly pathogenic avian influenza spreads in the USA. *Vet. Rec*. **176**, 505 (2015). 10.1136/vr.h2582 25977485

[6] USDA. Update on Avian Influenza Findings Poultry Findings Confirmed by USDA’s National Veterinary Services Laboratories. (2015).

[7] Ip HS (2015). Novel Eurasian highly pathogenic avian influenza A H5 viruses in wild birds, Washington, USA, 2014. Emerg. Infect. Dis..

[8] Torchetti MK (2015). Novel H5 Clade 2.3.4.4 reassortant (H5N1) virus from a green-winged teal in Washington, USA. Genome Announc..

[9] Nayak B (2009). Immunization of chickens with Newcastle disease virus expressing H5 hemagglutinin protects against highly pathogenic H5N1 avian influenza viruses. PLoS ONE.

[10] Khattar SK (2015). Mucosal immunization with Newcastle disease virus vector coexpressing HIV-1 Env and Gag proteins elicits potent serum, mucosal, and cellular immune responses that protect against vaccinia virus Env and Gag challenges. mBio..

[11] Kim SH, Paldurai A, Xiao S, Collins PL, Samal SK (2014). Modified Newcastle disease virus vectors expressing the H5 hemagglutinin induce enhanced protection against highly pathogenic H5N1 avian influenza virus in chickens. Vaccine.

[12] Wang J (2015). Generation and evaluation of a recombinant genotype VII Newcastle disease virus expressing VP3 protein of Goose parvovirus as a bivalent vaccine in goslings. Virus Res..

[13] Zhao W (2014). Newcastle disease virus (NDV) recombinants expressing infectious laryngotracheitis virus (ILTV) glycoproteins gB and gD protect chickens against ILTV and NDV challenges. J. Virol..

[14] Kanabagatte BM (2014). A recombinant Newcastle disease virus (NDV) expressing infectious laryngotracheitis virus (ILTV) surface glycoprotein D protects against highly virulent ILTV and NDV challenges in chickens. Vaccine.

[15] Huang Z, Elankumaran S, Yunus AS, Samal SK (2004). A recombinant Newcastle disease virus (NDV) expressing VP2 protein of infectious bursal disease virus (IBDV) protects against NDV and IBDV. J. Virol..

[16] Vigil A, Martinez O, Chua MA, Garcia-Sastre A (2008). Recombinant Newcastle disease virus as a vaccine vector for cancer therapy. Mol. Ther..

[17] Nakaya T (2001). Recombinant Newcastle disease virus as a vaccine vector. J. Virol..

[18] Liu Q (2015). Newcastle disease virus-vectored H7 and H5 live vaccines protect chickens from challenge with H7N9 or H5N1 avian influenza viruses. J. Virol..

[19] Schroer D (2011). Efficacy of Newcastle disease virus recombinant expressing avian influenza virus H6 hemagglutinin against Newcastle disease and low pathogenic avian influenza in chickens and turkeys. Avian Dis..

[20] Nagy A (2016). Recombinant Newcastle disease virus expressing H9 HA protects chickens against heterologous avian influenza H9N2 virus challenge. Vaccine.

[21] Ferreira HL (2014). Comparison of single 1-day-old chick vaccination using a Newcastle disease virus vector with a prime/boost vaccination scheme against a highly pathogenic avian influenza H5N1 challenge. Avian Pathol..

[22] Lardinois A (2012). Potency of a recombinant NDV-H5 vaccine against various HPAI H5N1 virus challenges in SPF chickens. Avian Dis..

[23] Ayllon J, Garcia-Sastre A, Martinez-Sobrido L (2013). Rescue of recombinant Newcastle disease virus from cDNA. J. Vis. Exp..

[24] Volkova MA (2014). Adjuvant effects of chitosan and calcium phosphate particles in an inactivated Newcastle disease vaccine. Avian Dis..

[25] Facon C, Guerin JL, Lacroix F (2005). Assessment of newcastle disease vaccination of houbara bustard breeders (Chlamydotis undulata undulata). J. Wildl. Dis..

[26] Ferreira HL (2012). Immune responses and protection against H5N1 highly pathogenic avian influenza virus induced by the Newcastle disease virus H5 vaccine in ducks. Avian Dis..

[27] Sarfati-Mizrahi D (2010). Protective dose of a recombinant Newcastle disease LaSota-avian influenza virus H5 vaccine against H5N2 highly pathogenic avian influenza virus and velogenic viscerotropic Newcastle disease virus in broilers with high maternal antibody levels. Avian Dis..

[28] DiNapoli JM (2007). Immunization of primates with a Newcastle disease virus-vectored vaccine via the respiratory tract induces a high titer of serum neutralizing antibodies against highly pathogenic avian influenza virus. J. Virol..

[29] Ge J (2011). Newcastle disease virus-vectored rabies vaccine is safe, highly immunogenic, and provides long-lasting protection in dogs and cats. J. Virol..

[30] Klimov A (2012). Influenza virus titration, antigenic characterization, and serological methods for antibody detection. Methods Mol. Biol..

[31] Reed L, Muench H (1938). A simple method of estimating fifty per cent endpoints. Am. J. Epidemiol..

[32] Krammer F, Palese P (2013). Influenza virus hemagglutinin stalk-based antibodies and vaccines. Curr. Opin. Virol..

