# Author Correction: Newcastle disease virus-based H5 influenza vaccine protects chickens from lethal challenge with a highly pathogenic H5N2 avian influenza virus

**DOI:** 10.1038/s41541-018-0054-8

**Published:** 2018-05-17

**Authors:** Jingjiao Ma, Jinhwa Lee, Haixia Liu, Ignacio Mena, A. Sally Davis, Sun Young Sunwoo, Yuekun Lang, Michael Duff, Igor Morozov, Yuhao Li, Jianmei Yang, Adolfo García-Sastre, Juergen A. Richt, Wenjun Ma

**Affiliations:** 1 Department of Diagnostic Medicine/Pathobiology, College of Veterinary Medicine, Kansas State University, Manhattan, KS USA; 2 Department of Microbiology, Icahn School of Medicine at Mount Sinai, New York, NY USA; 3 Global Health and Emerging Pathogens Institute, Icahn School of Medicine at Mount Sinai, New York, NY USA; 4 Innovation Team for Pathogen Ecology Research on Animal Influenza Virus, Department of Avian Infectious Disease, Shanghai Veterinary Research Institute, Chinese Academy of Agricultural Sciences, Shanghai, China; 5 Department of Medicine, Division of Infectious Diseases, Icahn School of Medicine at Mount Sinai, New York, NY USA; 6 Present Address: Shanghai Jiao Tong University, Shanghai, China

**Correction to:** *npj Vaccines* (2017) 10.1038/s41541-017-0034-4, Published online 4 December 2017

The wrong contract number was quoted in the Acknowledgments section of this Article. This work was supported by NIAID funded Centers of Excellence for Influenza Research and Surveillance under contract number HHSN272201400006C, not HHSN266200700006C as indicated in the original paper.

